# Analysis of Changes in Retinal Thickness in Type 2 Diabetes without Diabetic Retinopathy

**DOI:** 10.1155/2018/3082893

**Published:** 2018-02-25

**Authors:** Jing Jiang, Yan Liu, Yingchao Chen, Bo Ma, Yiwen Qian, Zhenzhen Zhang, Dongqing Zhu, Zhiliang Wang, Xiaofang Xu

**Affiliations:** ^1^Department of Ophthalmology, Ninth People's Hospital, Shanghai JiaoTong University School of Medicine, Shanghai, China; ^2^Department of Ophthalmology, Huashan Hospital of Fudan University, Shanghai, China; ^3^Shanghai Key Laboratory of Orbital Diseases and Ocular Oncology, Shanghai, China; ^4^Department of Endocrinology, Shanghai Ninth People's Hospital, Shanghai Jiaotong University School of Medicine, Shanghai, China

## Abstract

**Objective:**

To examine the changes in retinal thickness of patients with diabetes without DR.

**Designs:**

A randomization, crossover, retrospective practice.

**Participants:**

43 diabetic patients and 43 ethnic-, age-, and sex-matched controls.

**Methods:**

Full retinal thicknesses of ten areas were assessed using spectral domain optical coherence tomography. Confounding variables, such as age, gender, and glycated haemoglobin (HbA1c) level, were assessed by regression analysis.

**Main Outcome Measures:**

Mean retinal thickness of ten areas.

**Results:**

The mean thickness of the fovea was 215.8 ± 18.9 *μ*m in the diabetes group and 222.0 ± 18.6 *μ*m in the control group (*p* = 0.04). The mean thickness of the temporal parafovea was 319.9 ± 16.7 *μ*m in the diabetes group and 326.0 ± 14.4 *μ*m in the control group (*p* = 0.01). The mean thickness of the temporal perifovea was 276.4 ± 27.9 *μ*m in the diabetes group and 284.8 ± 17.4 *μ*m in the control group (*p* = 0.02). There were no significant differences in retinal thickness between groups in other areas (*p* > 0.05). Regression analysis revealed that decreased retinal thickness of the temporal perifovea was associated with a higher HbA1c level (>8.7%) (*p* = 0.04).

**Conclusion and Relevance:**

Subtle structural changes in the retina may occur in diabetes without DR. Decreased retinal thickness appeared to begin in the fovea and temporal areas. A high HbA1c level was the main factor influencing retinal thickness.

## 1. Introduction

Diabetes is a disease characterized by metabolic dysregulation. It occurs worldwide, and the number of people with diabetes is estimated to increase dramatically from 171 million in 2000 to 366 million in 2030 [1]. One-third of diabetic people develop some degree of diabetic retinopathy (DR), which has become the leading cause of vision loss in working-age adults [2]. It is important to detect the early signs of DR to facilitate timely monitoring and referral.

Clinical features of DR are undetectable at early stages. Traditional methods for evaluating DR, including slit-lamp biomicroscopy and stereo fundus photography, are relatively insensitive to small pathological changes in the retina. In addition, highly sensitive fluorescein angiography is invasive and not suitable for repeated examination. Optical coherence tomography (OCT) is a rapid, noninvasive, and useful imaging technology for cross-sectional and tomographic imaging in biological tissues, which is especially useful for quantitative and qualitative assessment of macula [3, 4]. OCT can provide objective documentation of retinal structural changes in eyes with DR even when the changes are not evident through slit-lamp biomicroscopy or angiography [5, 6].

Several studies have elucidated changes in the retinal thicknesses in patients with diabetes. Increases or decreases in retinal thickness have been reported in diabetes with or without DR, respectively, and various mechanisms have been proposed to be responsible for these changes [7–9]. The purpose of this study was to quantify the differences in retinal thickness between healthy individuals and individuals with diabetes without DR. In addition, our study evaluated the association of retinal thickness with age, gender, duration of diabetes, and glycated haemoglobin (HbA1c) levels.

## 2. Methods

This was a single-centre, retrospective cross-sectional case series performed at the Department of Ophthalmology, Ninth People's Hospital, Shanghai JiaoTong University, School of Medicine, from February 1, 2016, to July 15, 2016. The study was approved by the institute's ethics committee. Informed consent was provided by each subject for participation in the study.

### 2.1. Subjects

A total of 86 eyes of 43 patients with type 2 diabetes mellitus (group A) and the same number of samples in healthy subjects (group B, control group) were recruited. All participants underwent a comprehensive ophthalmologic examination consisting of visual acuity assessment (best corrected visual acuity [BCVA]), intraocular pressure (IOP) assessment, slit-lamp biomicroscopy combined with retinoscope, and fundus fluorescein angiography (FFA). The levels of peripheral fasting blood glucose (Portable Blood Glucose Meters; Johnson OneTouch Ultra; Johnson & Johnson Co, NJ, USA) and glycated haemoglobin A1C (HbA1c) were also simultaneously measured. The measurement method of HbA1c was high-pressure liquid chromatography (HPLC; Bio-rad Variant II; Bio-rad Co, CA, USA). This detection method is relatively accurate and stable, which has been widely used in routine screening for diabetes in China [10, 11].

For group A, an eye was eligible if it met the following criteria: (1) definite diagnosis of type 2 diabetes; (2) no DR (Early Treatment Diabetic Retinopathy Study level 10) on the basis of clinical exam and FFA; (3) no history of major ocular surgery or treatment, such as panretinal photocoagulation (PRP); (4) no macular pathology, such as epimacular membrane (EMM), vitreomacular traction (VMT), and age-related macular degeneration (AMD) found during clinical examination; and (5) no refractive errors or a refractive error less than ±3.0 D. For group B, the inclusion criteria were the same except that the subjects had no history of diabetes.

### 2.2. Optical Coherence Tomography Imaging

The retinal images were obtained using a Heidelberg Spectralis OCT instrument (wavelength: 870 nm; Heidelberg Engineering Co, Heidelberg, Germany) with version 6.0 software. Heidelberg Eye Explorer software (version 1.9.10.0, Heidelberg Engineering Co, Heidelberg, Germany) was used to perform measurements.

**Figure 1:**
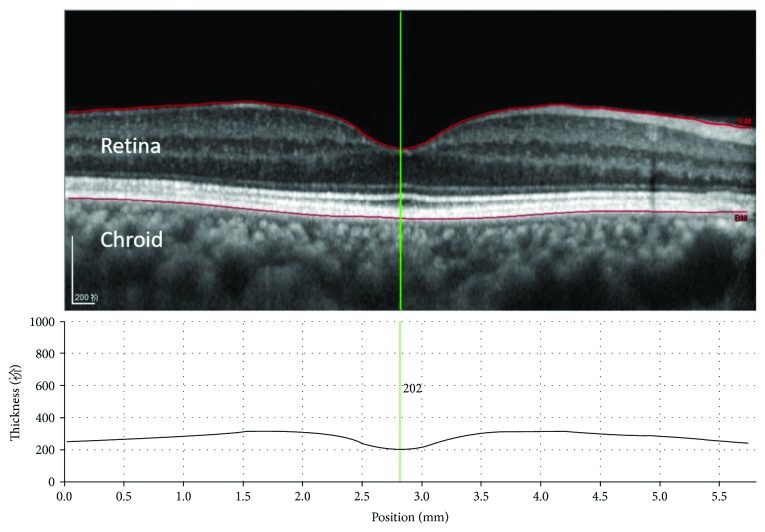
Enhanced depth imaging spectral domain optical coherence tomography (EDI-SD OCT) using Heidelberg Spectralis. The scan reveals normal retinal and choroidal anatomy. The foveal retinal thickness was determined on the basis of the distance between the two segments (red lines), which was measured in the “thickness profile” window by using the thickness diagram at the selected targets (the green line).

**Figure 2:**
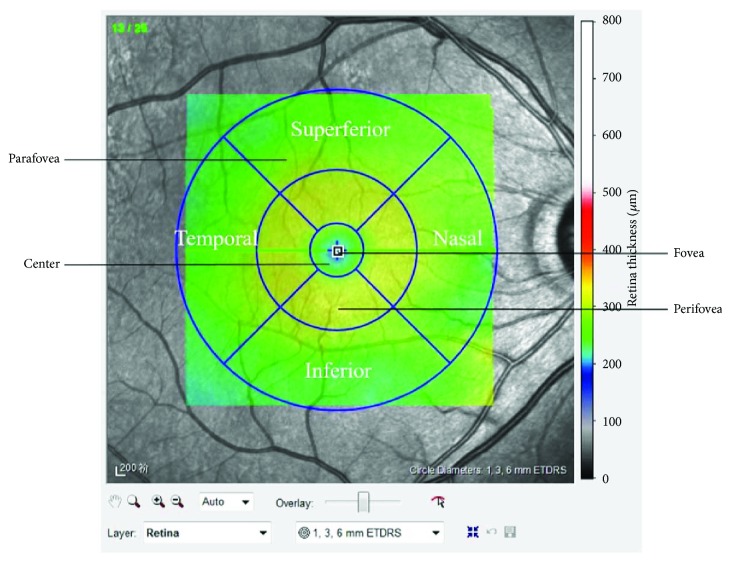
The ten areas examined included the fovea, central foveal circle (diameter = 1 mm), parafoveal circle (diameter = 3 mm), and perifoveal circle (diameter = 6 mm). The parafoveal area and the perifoveal area were further subdivided into superior, inferior, temporal, and nasal subfields.

Retinal thickness was defined as the height from the vitreoretinal interface to the retinal pigment epithelium (RPE) (Figure 1) [7, 12]. The macular region, with the exception of the fovea, was divided into three zones (centre, parafovea, and perifovea) with three concentric circles whose diameters were 1, 3, and 6 mm, respectively. Then, the parafovea and perifovea zones were further subdivided into four quadrants with two diagonal lines. Finally, in this study, a total of ten areas (fovea, centre, superior parafovea, superior perifovea, inferior parafovea, inferior perifovea, nasal parafovea, nasal perifovea, temporal parafovea, and temporal perifovea) were available for analysis. Firstly, the zones were partitioned automatically by using Stratus OCT software (version 6.0; Heidelberg Engineering Co, Heidelberg, Germany); then it would be artificially adjusted according to actual situation of the picture by the same examiner (Dr. Jing Jiang). A schematic of the examined zones is presented in Figure 2.

**Figure 3:**
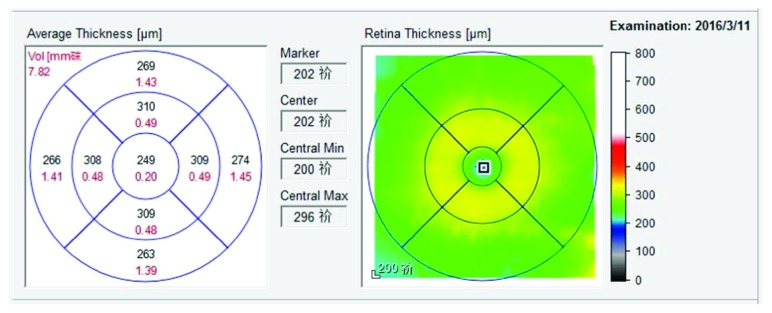
Retinal thickness map, in which averages of retinal thickness measurements of nine areas (except the fovea) are calculated and automatically displayed.

The retinal thickness was measured along 25 horizontal lines, each of which was 6 mm long and centred at the fovea. Thirty frames were averaged together with the aid of eye tracking, and images with a quality score ≥ 25 were selected. The lines crossing the foveal region were automatically calculated and converted to foveal thickness by using Stratus OCT software (version 6.0; Heidelberg Engineering Co, Heidelberg, Germany). For the remaining regions, average retinal thickness was calculated as the weighted average of all thickness measurements in the region (Figure 3). Data were then reassessed by two blinded independent examiners (Dr. Jing Jiang and Dr. Yan Liu). A third blinded investigator (Dr. Xiaofang Xu) made the final decision if disagreements and discrepancies in the assessments arose.

### 2.3. Statistical Analysis

Statistics were calculated using SPSS software (IBM SPSS, Version 22.0, IBM Corporation, Armonk, NY). Continuous variables, such as age, HbA1c level, and retinal thickness, are presented as the means ± SD. The chi-square test and two-sample *t*-test were performed to evaluate the differences between the study groups. The Pearson correlation coefficient was used to assess the correlation between any two nonnormally distributed variables. All *p* values reported were two-tailed, and *p* < 0.05 was considered to be statistically significant in this study.

## 3. Results

In total, 86 subjects (with 172 eyes) were recruited in this study. The average age of all subjects was 64.0 ± 7.0 years (range 45–80 years), and the male/female ratio was 32/54. A summary of the clinical parameters of participants in the two groups is provided in Table 1. Except for the history of diabetes and the higher HbA1c levels in group A, no further significant differences were observed between the two groups.

### 3.1. Changes in Retinal Thickness in Multiple Areas

Overall, the retinal thickness was thinnest in the foveal area, followed by the central area, in all subjects. The parafoveal areas were thicker than the perifoveal areas, and the nasal areas were thicker than the temporal areas. On average, the retinal thicknesses in all ten areas were thinner in group A than in group B. However, only the differences in the foveal, temporal parafoveal, and temporal perifoveal areas were statistically significant (*p* = 0.04, 0.01, and 0.02, resp.).  Table 2 presents a summary of the retinal thickness of the ten areas in both groups.

### 3.2. Factors Affecting the Thickness of the Retina

The duration of diabetes is a major factor that influences the progression of DR. In this study, the thickness of temporal areas showed a trend towards thinning with an increased duration of diabetes (*r* = −0.07, −0.20), but the differences were not statistically significant (*p* > 0.05).

The HbA1c level was another important factor affecting DR. To further explore the potential effect of HbA1c levels on retinal thickness, we divided the patients in group A into 2 subgroups according to their HbA1c levels. Group A1 (G_A1_) included subjects with an HbA1c level ≤ 8.7%, and group 2 (G_A2_) included subjects with an HbA1c level > 8.7%. After correction for age and sex, the Pearson correlation analysis revealed that only the temporal perifoveal thickness exhibited a negative correlation with HbA1c level (*r* = −0.31, *p* = 0.04), thus indicating that an increased HbA1c level contributed to decreased retinal thickness (Table 3).

## 4. Discussion

In this study, we examined retinal thickness in normal and type 2 diabetic individuals with no DR using SD-OCT and analyzed possible factors affecting retinal thickness. Our results indicated that thicknesses in the foveal and temporal (parafovea and perifovea) areas were significantly decreased in diabetic individuals with no visible sign of DR compared with normal controls. A thinning of the retina in the temporal perifovea area was associated with an increased HbA1c level (>8.7%).

In humans, diabetes can be classified into insulin-dependent diabetes mellitus (type 1 diabetes mellitus, T1DM) and noninsulin-dependent diabetes mellitus (type 2 diabetes mellitus, T2DM) according to pathogenesis [13]. Although the pathomechanism and prevalence populations of two types are totally different, retinal thickness and structure between T1DM and T2DM did not observe statistically significant difference after controlling for age, sex, duration of diabetes, and HbA1c levels. Generally speaking, T1DM has a prolonged course while T2DM is slightly older. Therefore, the potentially confounding effect of these variables may assess retinal thickness [14]. In our research, all patients were T2DM.

Changes in retinal thickness caused by diabetes are not fully understood. Previous studies have found decreased retinal thickness in diabetes with minimal or no DR compared with retinal thickness in nondiabetic individuals [15, 16]. In contrast, other researchers have observed a tendency towards increased retinal thicknesses in individuals with advanced DR [17, 18]. Our study provided an overall profile of retinal thickness in individuals with diabetes without detectable signs of DR. We observed that retinal thickness was commonly decreased in diabetes, especially in the foveal and temporal (parafovea and perifovea) areas. We believe that these conclusions that differ from those of the above studies may have been associated with differences in the type of diabetes or degree of DR in subjects, the scan protocols, the measuring instruments used, and the patients' age and sex. The decreased retinal thicknesses in individuals with early-stage diabetes reflected neurodegenerative changes, such as loss or degeneration of glial cells, in diabetic retinas. Srinivasan et al. have observed decreased thicknesses in the parafovea and perifovea in diabetic patients with peripheral neuropathy [19]. As the disease progressed, neurodegeneration may have caused vascular permeability, which may have led to the thickening of the retinal layers.

The pathological mechanisms of DR are complicated and multifactorial. Vascular dysfunction, the basic pathology underlying diabetes, can occur in early stages of DR, in the absence of structural and functional abnormalities of the retina. The fovea has the highest density of cones and therefore has an increased metabolic demand [20]. The temporal region has the thinnest retina [21]. We hypothesize that the foveal and temporal regions may therefore be more susceptible to microvascular or ischaemic insults because of the above structural features.

Previous studies have reported that several risk factors, including HbA1c level, duration of diabetes, hypertension, hyperlipidaemia, body mass index (BMI), sex, and insulin treatment, are involved in the development of DR. Among the factors above, a long duration of diabetes, high HbA1c levels, and insulin treatment are predictive factors for the deterioration of DR [22, 23]. To determine the cause of retinal thinning in this study, we analyzed the relationships among HbA1c level, duration of diabetes, and retinal thickness. Although the thickness of the temporal areas had a negative correlation with diabetes duration, the difference was not statistically significant. The reasons for this result may be that the course time provided by patients was the duration confirmed by diagnostic examination, which was often shorter than the actual duration of illness, and the so-called “course of diabetes” may not have been sufficient to lead to significative retinal structure damage.

HbA1c levels predict the incidence and progression of DR [24]. Retinal thickness is inversely correlated with HbA1c levels [25]. Another study has shown that a decrease in HbA1c is associated with thickening of inner retinal layers in the parafovea after bariatric surgery, which dramatically improves the metabolic profile in diabetes [26]. Hypoxic apoptosis of ganglion and axonal cells caused by the high affinity of HbA1c on oxygen (most oxygen molecule integrated with the elevated level of HbA1c instead of ganglion and axonal cells, which lead to the latter hypoxia) may be the most important underlying pathological mechanism. In this study, we also observed an inverse correlation between retinal thickness (temporal perifoveal area) and HbA1c levels. Our study indicated a strong correlation between DR and HbA1c.

In summary, we observed that retinal thickness in the foveal and temporal areas was decreased in diabetes without any clinical signs of DR, and a high HbA1c level was associated with a thinner retina in the temporal perifoveal area in this study. Our results suggest that the neurodegenerative damage of the retina may occur before vasculopathy in the very early stages of diabetes. SD-OCT is a valuable diagnostic tool for providing detailed measurements of the retina for detection of early-stage DR. The limitations of this study included the predominantly homogenous subject group (100% Asians from China), a self-reported duration of disease, and a relatively small sample size, which may have increased the bias of the study. Larger and longitudinal studies are necessary to verify these results.

## Figures and Tables

**Table 1:** Clinical characteristics of the two groups.

Clinical variables	Diabetes (no DR)	Control (normal)	*p* values
	Group A (*n* = 43)	Group B (*n* = 43)	
Age (years)	64.4 ± 8.4	63.7 ± 5.3	0.65
Male/female	17/26	15/28	0.66
History of hypertension	14/43	12/43	0.64
Duration of DM	13.3 ± 8.5	N/A	—
HbA1c level (%)	9.3 ± 2.2	5.3 ± 1.0	**<0.001**

DR: diabetic retinopathy; DM: diabetes mellitus; HbA1c: glycosylated haemoglobin.

**Table 2:** Retinal thickness of ten areas in the two groups (*μ*m, mean ± SD).

Retinal areas	Diabetes (no DR)	Control (normal)	*p* values
	Group A (*n* = 86)	Group B (*n* = 86)	
Fovea	215.8 ± 18.9	222.0 ± 18.6	**0.04**
Center	258.4 ± 23.0	261.3 ± 16.8	0.36
Superior parafovea	332.9 ± 18.6	337.3 ± 15.0	0.10
Superior perifovea	292.1 ± 17.5	296.0 ± 14.6	0.12
Inferior parafovea	328.6 ± 16.4	333.0 ± 16.7	0.09
Inferior perifovea	279.4 ± 17.2	282.9 ± 15.1	0.17
Nasal parafovea	334.3 ± 18.5	338.2 ± 15.8	0.15
Nasal perifovea	307.4 ± 19.6	311.8 ± 17.4	0.13
Temporal parafovea	319.9 ± 16.7	326.0 ± 14.4	**0.01**
Temporal perifovea	276.4 ± 27.9	284.8 ± 17.4	**0.02**

SD: standard deviation; DR: diabetic retinopathy.

**Table 3:** Association between HbA1c levels and regions.

Retinal areas	Diabetes group only			
	G_A1_		G_A2_	
	*r*	*p*	*r*	*p*
Fovea	−0.15	0.35	−0.30	0.09
Temporal parafovea	−0.16	0.33	−0.12	0.06
Temporal perifovea	−0.09	0.57	−0.31	0.04

HbA1c: glycosylated haemoglobin.
